# Dose Escalation of Adalimumab in Patients with Hidradenitis Suppurativa: A Retrospective Case Series

**DOI:** 10.1177/12034754241308248

**Published:** 2025-01-07

**Authors:** Nicolas Caudrelier, Hélène Veillette

**Affiliations:** 1 Division of Dermatology, Department of Medicine, CHU de Québec-Université Laval, Québec, QC, Canada

**Keywords:** hidradenitis suppurativa, adalimumab, tumor necrosis factor inhibitor, therapeutic drug monitoring, anti-adalimumab antibodies

## Abstract

**Background:**

Adalimumab is a central treatment for moderate-to-severe hidradenitis suppurativa (HS). However, half of patients treated with adalimumab for HS do not achieve a clinically significant response. Data on non-immunological factors correlating clinical response and adalimumab blood concentration are scarce.

**Objectives:**

To determine whether increasing adalimumab dose to 80 mg weekly can improve the clinical outcome of patients unresponsive to adalimumab 40 mg weekly. To identify parameters influencing disease activity and those modifying serum adalimumab concentration.

**Methods:**

This is a retrospective case series from a tertiary dermatology clinic, comprising 40 patients with moderate-to-severe HS with suboptimal response to the FDA-approved dose of adalimumab. All patients had a measurement of blood adalimumab concentration. Depending on their dosage, some patients had their adalimumab dose increased to 80 mg weekly while others stayed at 40 mg dose weekly. Chi-squared and ANOVA tests were used for data analysis.

**Results:**

43.8% of patients who increased to a weekly dose of 80 mg clinically improved at their follow-up, compared with 33.3% of those who stayed at a weekly dose of 40 mg. The dose increase led to a statistically significant increase in serum concentration of adalimumab (*P* < .001). Higher serum adalimumab concentration is associated with lower disease activity (*P* = .043). Normal-weight patients had significantly higher concentrations than overweight patients (*P* = .011).

**Conclusions:**

An increase in the weekly dose of adalimumab led to a nonstatistically significant improvement in clinical response but led to a statistically significant increase in serum concentration.

## Introduction

Hidradenitis suppurativa (HS) is a chronic inflammatory dermatosis of the pilosebaceous units, which mainly affects the intertriginous areas. HS manifests as inflamed nodules, abscesses, cutaneous sinuses, and scarring, which are associated with pain, malodorous discharge, significant psychological burden, and decreased quality of life. The prevalence is estimated to be between 1 and 4% of the general population, being more frequent in young adults, women, and people of African descent.^1^

The pathogenesis of HS is not completely understood, yet the main cytokines involved are tumor necrosis factor alpha (TNF-α) and interleukin 17.^1^ Despite improved understanding of the pathophysiology of HS, optimizing treatment remains an ongoing challenge.

Adalimumab is a fully human monoclonal antibody capable of inhibiting TNF-α. Randomized phase 3 clinical trials have demonstrated the effectiveness of adalimumab in the treatment of HS.^2^ Adalimumab was approved by Health-Canada in 2016 in cases of moderate-to-severe HS for patients who have not responded to conventional therapy, including systemic antibiotics. However, approximately half of patients receiving adalimumab for HS do not achieve a significant clinical response.^3^ It is assumed that patients who do not respond sufficiently to adalimumab have non-immunological pharmacokinetic factors (such as high BMI and degree of systemic inflammation), or immunological factors [such as anti-adalimumab antibodies (AAAs)], which interfere with the drug’s functioning.^3^ It has been shown that in other inflammatory conditions (rheumatoid arthritis, Crohn’s disease), therapeutic drug monitoring, consisting of measuring serum drug dosage and anti-drug antibodies, can improve clinical response.^4,5^ Abdalla et al. demonstrated that patients suffering from HS and who developed AAAs have significantly lower blood levels of adalimumab than patients who did not develop AAAs and that 89% of them are suboptimal responders.^5^ Other studies have demonstrated that increasing the administered dose of adalimumab to 80 mg weekly may improve clinical response, regardless of AAA status or serum adalimumab levels.^6,7^ However, limited information has yet been published to determine whether an increase in the dose of adalimumab can improve the clinical response in patients who have a suboptimal response to the usual dose of adalimumab, who have not developed AAAs and who have subtherapeutic serum adalimumab dosage.

## Patients

This retrospective case series comprises 40 patients suffering from moderate-to-severe HS, with suboptimal response to adalimumab at an initial dose of 40 mg per week and who have undergone therapeutic drug monitoring, between January 2020 and April 2024 at the CHU de Québec-Université Laval (Quebec City, Canada). Patients with adequate response to adalimumab at a dose of 40 mg weekly were not included. This study was accepted by the ethics committee of the CHU de Québec. Data were obtained by review of medical records. Patient demographics are described in Supplemental Table S1. Patient age ranged from 19 to 69 years (mean: 40.8). Most patients are women (77.5%). 57.5% of patients have Hurley stage II and 40% have Hurley stage III. 45% of patients are smokers. 77.5% of patients have a BMI greater than 25. The most frequent comorbidities are from most to less frequent: acne, pilonidal sinus, diabetes, high blood pressure, and anxio-depressive disorder. Patients had tried on average more than five other treatments, among which the most common were doxycycline, intralesional triamcinolone acetonide, metformin, and minocycline. Topical treatments were not considered in the current study.

## Methods

Disease activity is assessed by the clinician according to the Hidradenitis Suppurativa Physician Global Assessment (HS-PGA) after a minimum of 12 weeks of treatment with adalimumab at a fixed dose. Patients were divided into three groups: low activity (HS-PGA = 1 or 2, ie, without active lesions), moderate activity (HS-PGA = 3 or 4, ie, up to 5 abscesses or fistulas, and up to 9 active nodules), and high activity (HS-PGA of 5 or 6 ie, 6 or more abscesses or fistulas or 10 or more active nodules).^8^ An improvement in clinical response is considered to be achieved if a patient is deemed to move from one category of disease activity to one of less severity. For example, a patient is considered to have an improvement in clinical response if his disease activity changes from moderate to low activity, or from high to moderate or low activity. The adalimumab serum trough levels and AAA levels were measured after a minimum of 12 weeks of fixed dose adalimumab treatment. Blood was collected within 4 days before the next injection.

Several comparisons were made in this study. Regarding the improvement in the clinical response at the follow-up, the group of patients remaining at a dose of 40 mg adalimumab per week (n = 12) was compared with the group of patients increased to a weekly dose of 80 mg (n = 16) (Supplemental Table S2). The patients who had an improvement in clinical response, and those who did not have clinical improvement in the two groups were also compared (Supplemental Table S2). Specifically for the group of patients increased to 80 mg, the pre- and post-dose increase serum dosages were compared (Supplemental Table S2). Then, to identify the factors affecting disease activity, all patients (n = 40) were compared irrespective of the administered dose (Supplemental Table S3). Finally, to determine the parameters influencing the serum concentration of adalimumab, all dosages (n = 63) were compared (Supplemental Table S4).

Descriptive statistics are used for data presentation: number and percentage of patients for categorical variables and number and mean for continuous variables. Comparisons of clinical values between different groups were examined using the chi-squared test for categorical variables or ANOVA for continuous variables. A *P*-value < .05 was considered statistically significant.

## Results

The primary objective of this study was to determine whether a dose increase in adalimumab to 80 mg per week can improve the clinical response of patients who do not have a satisfactory response on adalimumab 40 mg per week compared with patients remaining at a dose of 40 mg of adalimumab per week (Supplemental Table S2). Patients who had developed AAAs were excluded from our study. Among the 40 identified patients, 12 patients had to be excluded from this analysis: Six patients were not clinically evaluated after their dosage, two patients prematurely stopped adalimumab (one patient developed AAAs and one patient developed paradoxical psoriasis), and four patients already had a dose of 80 mg per week before dosing. Therefore, 26 patients were evaluated, of which 12 patients remained on a weekly dose of 40 mg adalimumab, and 16 patients had a weekly increased dose of 80 mg adalimumab. In the group of patients remaining on 40 mg of adalimumab, four patients (33.3%) had an improvement in clinical response at their medical follow-up. The mean adalimumab serum concentration of this entire group is 17.3 µg/mL, the concentration of those who have not improved clinically at the follow-up is 15.5 µg/mL, and the concentration of those who improved is 18.1 µg/mL. In the group of 16 patients switched to an increased weekly dose of 80 mg of adalimumab, seven improved (43.8%, *P* = .576). Their average dosage before the dose increase is 7.8 µg/mL, and their average dosage after the dose increase is 18.7 µg/mL (*P* < .001). In this group, after the weekly dose increase to 80 mg, the average dosage of patients who did not have an improvement in clinical response is 18.6 µg/mL (*P* = .080) while it is 18.8 µg/mL for patients who had clinical improvement (*P* = .897).

The secondary objectives of this study were to determine the parameters influencing disease activity (Supplemental Table S3) and the parameters influencing serum adalimumab concentration (Supplemental Table S4). To maximize the analyzes on the serum concentration of adalimumab, all the evaluations and dosages available in all patients were used and considered independently; therefore, certain patients could be included several times. A total of 63 measurements are available to evaluate the parameters influencing the dosage. For the analyzes on the parameters influencing disease activity, all patients were considered (n = 40).

Parameters influencing disease activity are presented in Supplemental Table S3. A higher serum concentration of adalimumab is associated with lower activity (*P* = .043). Parameters not influencing disease activity are age (*P* = .398), sex (*P* = .148), and BMI (*P* = .148).

Parameters influencing serum concentration of adalimumab are presented in Supplemental Table S4. Regarding the administered dose, patients receiving a weekly dose of 40 mg have a mean concentration of 12.6 µg/mL while patients receiving a weekly dose of 80 mg had a mean concentration of 16.9 µg/mL (*P* = .071). Age (*P* = .117), sex (*P* = .148), and smoking status (*P* = .519) do not influence serum concentration of adalimumab. Relative to BMI, normal-weight patients have a higher average concentration than overweight or obese patients (*P* = .011).

## Discussion

Adalimumab is a central treatment for moderate-to-severe HS. It is therefore important to determine the factors that may influence a suboptimal response to this treatment, and how to optimize patient responses. Studies have looked at the role of AAAs,^3^ but little is known about the factors influencing the response for patients who have not developed AAAs. This pilot study on 40 patients identifies several of these factors, namely, serum adalimumab concentration and BMI.

Among patients (n = 16) who were followed long enough to determine whether a dose increase in adalimumab to 80 mg per week from 40 mg per week led to a clinical response, 43.8% improved at the follow-up compared with only 33.3% for patients remaining at a dose of 40 mg weekly (n = 12). The number of patients with clinical improvement is nonstatistically increased by 31.3% (*P* = .576) in the 80 mg per week group, compared with the group remaining at 40 mg. The mean initial adalimumab serum concentration of patients who were increased to a weekly dose of 80 mg was significantly lower than the mean concentration of those who remained at a weekly dose of 40 mg (7.5 µg/mL vs 17 .3 µg/mL; *P* < .001). Increasing the adalimumab dose to 80 mg weekly also led to a statistically significant increase in serum concentration (18.7 µg/mL; *P* < .001).

Regarding factors influencing disease activity, in our study, including all 40 patients, higher serum adalimumab concentration is significantly associated with lower activity (*P* = .043). Serum concentration of adalimumab would possibly be a biological parameter to take into consideration to optimize the response of patients who have not developed AAAs. The other associations made are not statistically significant in this retrospective series. Still, while the small population did not reveal statistically significant differences, factors associated with lower disease activity were age 30 and over, male gender, active smoking, and a normal range BMI. It is surprising that in this population, nonsmoking patients had a higher disease activity (47.1% compared to 16.7% for smokers, *P* = .143) even though smoking is an established risk factor for HS.^9^

Regarding factors influencing serum adalimumab concentration, normal range BMI patients had significantly higher concentrations than overweight or obese patients (19.3 µg/mL vs 13.0 µg/mL; *P* = .011). This may be caused by a higher volume of distribution and increased excretion.^10^ Other nonstatistically significant observations indicate that the serum concentration of adalimumab is higher with higher administered doses, in older patients, in women, and in active smokers.

Limitations of this study are the small sample size and its retrospective nature. Due to the small number of patients included, most of our results failed to reach statistical significance. Furthermore, HS is a disease of alternating phases of flare-ups and remissions. Thus, patients can improve spontaneously upon the follow-up. Besides, we combined obese and overweight patients together, whereas several studies have demonstrated that only obesity influences HS.^11^ In addition, patients received other topical and systemic treatments, which may have influenced the results. This study has an overrepresentation of patients with an inadequate response to adalimumab. Given the scarcity of data in the literature, this study remains very relevant, and prospective and randomized studies on larger populations should be conducted to confirm these results.

In conclusion, in our small population of patients with a suboptimal response to adalimumab for HS, who did not developed AAAs and with a subtherapeutic adalimumab dosage, an increase in the weekly dose of adalimumab from 40 to 80 mg led to a nonstatistically significant improvement in clinical response. This increase in administered dose also led to an increase in the serum concentration of adalimumab. The serum concentration of adalimumab significantly influenced disease activity in our population of suboptimal adalimumab responders. BMI significantly influenced serum concentration. Further studies are needed to identify whether an increase in adalimumab dose can significantly improve clinical response, and to identify factors influencing disease activity and adalimumab concentration.

## Supplemental Material

sj-docx-1-cms-10.1177_12034754241308248 – Supplemental material for Dose Escalation of Adalimumab in Patients with Hidradenitis Suppurativa: A Retrospective Case Series Supplemental material, sj-docx-1-cms-10.1177_12034754241308248 for Dose Escalation of Adalimumab in Patients with Hidradenitis Suppurativa: A Retrospective Case Series by Nicolas Caudrelier and Hélène Veillette in Journal of Cutaneous Medicine and Surgery

sj-docx-2-cms-10.1177_12034754241308248 – Supplemental material for Dose Escalation of Adalimumab in Patients with Hidradenitis Suppurativa: A Retrospective Case Series Supplemental material, sj-docx-2-cms-10.1177_12034754241308248 for Dose Escalation of Adalimumab in Patients with Hidradenitis Suppurativa: A Retrospective Case Series by Nicolas Caudrelier and Hélène Veillette in Journal of Cutaneous Medicine and Surgery
