# Supplementary material for: Dose Escalation of Adalimumab in Patients with Hidradenitis Suppurativa: A Retrospective Case Series
Source: J Cutan Med Surg. 2025 Jan 7;29(3):250–3. doi: 10.1177/12034754241308248 (PMC12171030; doi:10.1177/12034754241308248)
Supplement: sj-docx-1-cms-10.1177_12034754241308248 – Supplemental material for Dose Escalation of Adalimumab in Patients with Hidradenitis Suppurativa: A Retrospective Case Series [file sj-docx-1-cms-10.1177_12034754241308248.docx]

Figure S1. Study schematic
