# Supplementary material for: Dose Escalation of Adalimumab in Patients with Hidradenitis Suppurativa: A Retrospective Case Series
Source: J Cutan Med Surg. 2025 Jan 7;29(3):250–3. doi: 10.1177/12034754241308248 (PMC12171030; doi:10.1177/12034754241308248)
Supplement: sj-docx-2-cms-10.1177_12034754241308248 – Supplemental material for Dose Escalation of Adalimumab in Patients with Hidradenitis Suppurativa: A Retrospective Case Series [file sj-docx-2-cms-10.1177_12034754241308248.docx]

**Supplemental Tables**

Table S1. Demographic characteristics

| **Characteristic** | **Percentage** | **Value (n=40)** |
| --- | --- | --- |
| **Age (years)** |  | 40.8 (19–69) |
| **Sex** |  |  |
| Female | 77.5% | 31 |
| Male | 22.5% | 9 |
| **Hurley stage** |  |  |
| Hurley II | 57.5% | 23 |
| Hurley III | 40.0% | 16 |
| Unknown Hurley stage | 2.5% | 1 |
| **Smoking status** |  |  |
| Non-smoker | 42.5% | 17 |
| Smoker | 45.0% | 18 |
| Unknown smoking status | 12.5% | 5 |
| **BMI** |  |  |
| Normal weight (18.5-24.9) | 22.5% | 9 |
| Overweight or obese (>=25.0) | 77.5% | 31 |
| **Comorbidity** |  |  |
| Acne | 32.5% | 13 |
| Pilonidal sinus | 22.5% | 9 |
| Dissecting cellulitis of the scalp | 2.5% | 1 |
| High blood pressure | 12.5% | 5 |
| Dyslipidemia | 7.5% | 3 |
| Diabetes | 15.0% | 6 |
| Polycystic ovary syndrome | 5.0% | 2 |
| Crohn's disease | 7.5% | 3 |
| Anxio-depressive disorder | 12.5% | 5 |
| **Other medications tried** |  |  |
| Doxycycline | 82.5% | 33 |
| Minocycline | 50.0% | 20 |
| Clindamycin | 32.5% | 13 |
| Clindamycin and rifampicin | 27.5% | 11 |
| Cefadroxil or cefalexin | 25.0% | 10 |
| Amoxicillin/clavulanic acid | 22.5% | 9 |
| Trimethoprim/sulfamethoxazole | 30.0% | 12 |
| Ertapenem | 27.5% | 11 |
| Metformin | 52.5% | 21 |
| Isotretinoin | 25.0% | 10 |
| Spironolactone | 30.0% | 12 |
| Combined oral contraceptive pill | 22.5% | 9 |
| Intralesional triamcinolone acetonide | 67.5% | 27 |
| Prednisone | 20.0% | 8 |

Table S2. Improvement of clinical response and serum concentration of adalimumab depending on dose. *: 12 patients not evaluable

| **Population (n=40*)** | **Patients remaining on 40 mg (n=12)** | **Patients increased to 80 mg (n=16)** | **P value** |
| --- | --- | --- | --- |
| **Improvement of clinical response at follow-up** | 33.3% (4) | 43.8% (7) | 0.576 |
| **Serum concentration (µg/mL)** |  |  |  |
| Before dose increase | 17.3 | 7.8 | **<0.001** |
| After dose increase | ND | 18.7 (pre vs post dose increase p<0.001) |  |
| Patients who did not improve response | 15.5 (8) | 18.6 (9) | 0.080 |
| Patients who improved response | 18.1 (4) | 18.8 (7) | 0.897 |

Table 3. Parameters influencing disease activity (n=40 patients)

| **Characteristic** | **Low activity (n=3)** | **Moderate activity (n=24)** | **High activity (n=13)** | **P value** |
| --- | --- | --- | --- | --- |
| **Serum concentration (µg/mL)** | 22.4 (IC95% 9.1-35.6) | 13.6 (IC95% 11.7-15.5) | 13.0 (IC95% 8.1-18.0) | **0.043** |
| **Age** |  |  |  | 0.398 |
| 18–29years | 14.3% (1) | 28.6% (2) | 57.1% (4) |  |
| 30–49years | 4.3% (1) | 65.2% (15) | 30.4% (7) |  |
| 50–69years | 10.0% (1) | 70.0% (7) | 20.0% (2) |  |
| **Sex** |  |  |  | 0.148 |
| Female | 3.2% (1) | 64.5% (20) | 32.3% (10) |  |
| Male | 22.2% (2) | 44.4% (4) | 33.3% (3) |  |
| **Smoking status** |  |  |  | 0.143 |
| Non-smoker | 5.9% (1) | 47.1% (8) | 47.1% (8) |  |
| Smoker | 5.6% (1) | 77.8% (14) | 16.7% (3) |  |
| **BMI** |  |  |  | 0.148 |
| Normal weight (18.5-24.9) | 22.2% (2) | 44.4% (4) | 33.3% (3) |  |
| Overweight or obese (>=25.0) | 3.2% (1) | 64.5% (20) | 32.3% (10) |  |

Table S4. Parameters influencing serum adalimumab concentration (n=63 assays)

| **Characteristic** | **Mean serum concentration (µg/mL)** | **95% confidence interval** | **P value** |
| --- | --- | --- | --- |
| **Weekly dose administered** |  |  | 0.072 |
| 40 mg | 12.6 | 10.2-14.9 |  |
| 80 mg | 16.9 | 13.6-20.3 |  |
| **Age** |  |  | 0.117 |
| 18–29 years | 12.2 | 8.2-16.2 |  |
| 30–49 years | 13.3 | 10.4-16.1 |  |
| 50–69 years | 17.4 | 13.7-21.1 |  |
| **Sex** |  |  | 0.197 |
| Female | 14.9 | 12.5-17.3 |  |
| Male | 12.2 | 9.0-15.4 |  |
| **Smoking status** |  |  | 0.519 |
| Non-smoker | 13.4 | 10.6-16.1 |  |
| Smoker | 15.6 | 12.2-18.9 |  |
| **BMI** |  |  | **0.011** |
| Normal weight (18.5-24.9) | 19.3 | 13.4-25.3 |  |
| Overweight or obese (>=25.0) | 13 | 11.0-14.9 |  |
